# Coupling Coordination Measurement and Evaluation of Urban Digitalization and Green Development in China

**DOI:** 10.3390/ijerph192215379

**Published:** 2022-11-21

**Authors:** Siliang Guo, Yanhua Diao, Junliang Du

**Affiliations:** 1 School of Economics and Management, Qilu Normal University, Jinan 250200, China; 2 School of Economics and Management, Nanjing University of Aeronautics and Astronautics, Nanjing 211106, China; 3 School of Economics and Management, Shandong Youth University of Political Science, Jinan 250103, China

**Keywords:** urban digitalization, green development, coupling coordination, regional differences, evolution trend, spatial effect

## Abstract

The coordinated promotion of urban digitalization and green development is an inevitable requirement for sustainable development in the digital age. Based on the coupling mechanism of urban digitalization and green development, in this study, we took 282 cities at the prefecture level and above in China from 2011 to 2019 as the research object, and we constructed the evaluation index system and calculated the coupling coordination degree (CD&GDD) of the two through the coupling coordination degree model. We further used the Dagum Gini coefficient, kernel density estimation, Markov chain and Moran’s I to assess the spatial effects of the regional differences, dynamic evolution trends and degree of coupling coordination. The results show the following: (1) The level of urban digitalization and green development show a fluctuating upward trend, and the interaction between the two is obvious. (2) Although the CD&GDD of most cities is continuously improving, it is still at a low level. There are large differences in the levels between the regions. (3) The inter-regional differences are the main source of the large overall differences in the CD&GDD in China, and these are mainly composed of the hypervariable density and net differences between the regions. (4) The phenomenon of “club convergence” exists in the CD&GDD. (5) The coupling coordination relationship between cities has a substantial spatial effect, and the spatial effect has obvious regional heterogeneity. The results and conclusions provide a reference for developing countries to promote green and low-carbon urban development.

## 1. Introduction

In the context of low-carbon globalization, green development has become an international focus. Green development is a current mode of development featuring resource conservation, environmental friendliness and social progress, which can realize ecological benefits and guarantee economic and social benefits, including the three core elements of “economic development, resource conservation and environmental protection, and social welfare enhancement”, and pay more attention to the unity and coordination of economic development, social progress and ecological construction [1]. Countries and regions have stepped up their investments in green development, focusing on carbon neutrality and clean energy and actively shaping the capacity building and competitive advantages for green development. Currently, green development is an inevitable choice for the high-quality development of Chinese cities, and it is also the only way to meet people’s growing demands for a better ecological environment [1]. Urban digitalization has become a dominant trend in the economic and social development of various countries. It refers to the use of 5G, big data, artificial intelligence and other digital technologies to focus on the key areas and core links of urban operations, and to promote the comprehensive remodeling of economic society and urban governance [2], which is the key for cities to build core competitiveness in the digital revolution. China also regards digitalization as an important focus for high-quality urban development. The outline of China’s 14th Five-Year National Economic and Social Development Plan precisely proposes the acceleration of digital development and the building of a digital China. At the same time, it has made a series of strategic arrangements to create new advantages in the digital economy, accelerate the construction of a digital society, raise the level of digital government construction and create a sound digital ecology. Shanghai, Beijing and other local governments have also issued policies related to urban digital transformation, such as Shanghai’s Opinions on Comprehensively Promoting Urban Digital Transformation in Shanghai, and Beijing’s Implementation Plan on Accelerating the Construction of a Global Digital Economy Benchmark City. Green development emphasizes both environmental protection and efficiency, while digital technology can considerably improve efficiency on the premise of ensuring environmental protection by fostering a more flexible and efficient new economy, new forms of business and new driving forces [3]. Therefore, it is of great theoretical and practical importance to accurately understand the coordination relationship between urban digitalization and green development (CD&GDD), and to quantitatively analyze it, which has a certain reference value for the formulation of urban development policies and the implementation of differentiation strategies.

The existing independent studies on urban digitalization and green development are rich, while studies on the relationship between the two are relatively lacking. In terms of the research content, scholars have focused on the following: the measurement and spatial–temporal evolution characteristics of the digital economy development [4,5]; the dynamic mechanism of green development and the spatial–temporal differences [6,7]; the relationship between digital development and the ecological environment [8,9]; and the relationship between digitalization and high-quality development [10,11]. In recent years, some scholars have begun to consider the impact of the digital economy on green development [12,13]. In terms of the research scale, in the existing literature, researchers mainly focus on the macroscale national and provincial levels [14,15] and the microscale firm level [16,17]. Overall, in the existing research, the main focus has been on the impact of digitalization on green development, but there has been little analysis of the coordination between the two, let alone quantitative analyses of the interaction between them. In addition, in the existing analyses of the relationship between digitalization and green development, researchers often regard the research objects as independent individuals and only focus on the influence of internal factors. They fail to consider the dynamic study of the time, space and geographical factors on the coordinated development and evolution process at the city level at the same time, and they ignore the spatial effect of adjacent cities.

To this end, based on the coupling coordination mechanism between urban digitalization and green development, in this study, we used a variety of methods to measure and evaluate the degree of the coupling coordination between the two, as well as the regional differences and their dynamic evolution characteristics, in China and its four economic zones from the point of view of the time, space and geography of the evolution of neighboring systems, with the aim of providing scientific support for urban digital transformation and high-quality green development in China and other developing countries around the world. The main contributions of this paper are as follows: (1) We expand the theoretical mechanism of the relationship between urban digitalization and green development. Based on the existing literature, we provide an in-depth analysis of the two-way interaction coupling mechanism between urban digitalization and green development, which constrain and promote each other. Expanding on the existing research, we innovatively focus on the coupling coordination relationship between digitalization and green development, and we provide theoretical support for promoting the coordinated development of the two. (2) We explore the synergistic relationship between urban digitalization and green development from a systemic perspective. Taking the city as the research scale, we conducted a dynamic study on the evolution process of the synergistic relationship between urban digitalization and green development and the spatial effect of adjacent cities from the time, space and geographical elements, improving the shortcomings of the existing literature, in which researchers only focus on the impact of internal elements. In addition, in the existing empirical studies, the researchers mainly use China’s inter-provincial data as the study sample; however, there are still large gaps between the urban digitalization and green development in the different cities within a province. Moreover, by using Chinese city data as a sample, we could effectively expand the sample size, reduce the measurement errors and improve the accuracy of the analysis. (3) We provide empirical evidence on the coupling and synergy between urban digitalization and green development, which the existing research lacks. In this paper, we provide comprehensive measurements of the urban digitalization and green development, as well as the CD&GDD, and we analyze the regional differences in the CD&GDD using the Dagum Gini coefficient and its decomposition. We used kernel density estimates and Markov transition probability matrices to analyze the evolution trend of the CD&GDD from a time perspective, and we then used Moran’s I and the LISA test to analyze the spatial effects of the CD&GDD, which also deepen the coupling coordination mechanism between urban digitalization and green development.

## 2. Literature Review

The research on green development is extensive. The research content related to this paper mainly concerns evaluation measures of green development and its impact factors. First, the research content of green development mainly focuses on its evaluation index system construction and measurement analysis. Most of the existing studies have improved upon previous results. Among them, the three-system framework model of economy, society and environment, established by the United Nations Commission on Sustainable Development, is a relatively comprehensive system of evaluation indicators for green development at the present stage. Based on this, many scholars have conducted research on green economy accounting and green development capacity measurement. For example, Nicolas et al. [18] incorporated the three systems of environment, society and economy into the accounting framework, and finally evaluated the green and sustainable development ability of the country’s urban areas. The measurement of green development covers a wide range of areas, involving different geographical spaces and different industries. Geographically, the study covers all countries over the world [19,20,21], the Belt and Road [22] and OECD economies [23,24], as well as a certain country and its internal regional provinces [25,26]. In terms of industry, studies on the measurement of green development mainly focus on industry [27,28], particularly the manufacturing industry [29,30]. Secondly, the research content of green development involves the influence mechanism and promotion path of green development. The study focused on three areas. (1) The government management system. Lorek and Spangenberg (2014) [31] advocate that modernizing a social system can reduce the consumption of energy and other resources, which is conducive to green development. Dulal et al. (2015) [32] found that a government management system or policy innovation, especially fiscal policy innovation, is conducive to improving the local green development level. At the same time, they found that Asia, which has a higher rate of policy innovation, has a better state of green development. (2) Technological innovation. Samad and Manzoor (2015) [33] and Walz et al. (2017) [34] all agree that green technology innovation is the key and core factor for green growth and sustainable development. Whether in newly industrialized countries or traditional OECD countries, technological innovation capacity is a booster for the green development of countries. (3) Industrial development. Kim et al. (2014) [35] selected the role of South Korea’s industries in its green development. Shironitta (2016) [36] further compared the impact of the industrial structure of 40 countries on the green development of their economies. However, most studies agree that a country’s industrial structure is a key factor in determining its level of green development.

In the related research on the relationship between digitalization and green development, researchers have focused on the impact of the former on the latter. Most researchers have explored the impact of digitalization on green development at the macroscopic national or provincial levels. Digitalization has a positive impact on green development. Digitalization is environmentally friendly, and the information technology innovation that it triggers provides a more efficient way for the world to exchange information [37], which accelerates the transformation of the economic structure and meets the requirements of green development. Digital technology can optimize the industrial structure and improve energy efficiency [9], break through the inherent materialization of capital and reduce the demand for corporate land [38], and build a digital public platform to share the concept and way of green life with residents [1]. These have a “green effect,” which is conducive to low-carbon, healthy and sustainable economic development. Chen [39] took the BRICS countries as samples to empirically test the impact of digitalization on carbon emissions and found that digital technology substantially reduces carbon emissions in both the short and long term. After conducting case studies on cities on various continents, Balogun et al. [40] also concluded that digital technology has a positive impact on sustainable development. In addition, the development of digitalization can not only improve the productivity of traditional industries by promoting technological innovation [41], but it can also be an essential way to promote the integrated development of the manufacturing and service industries [42]. On the one hand, digitalization promotes the deconstruction and reorganization of the existing industrial chain. Digitization is safer and more stable and efficient than the traditional industrial chain [43], and it provides a fresh idea and feasible path for green development. On the other hand, digitalization is not conducive to regional green development. As an energy-intensive industry, the digital industry needs to pay attention to its inherent energy consumption [44]. The construction of digital infrastructure will also increase the consumption of electricity and additional energy sources [45]. Several researchers have also explored the impact of digitalization on green development from the microbusiness level. Enterprise digitization reduces the cost of information analysis and process optimization, which effectively improves the resource utilization of enterprises so that they can achieve higher levels of innovation output performance; these positive signals are critical factors for attracting external investors [46]. The easing of financing pressures further encourages enterprises to take on more social responsibility and engage in more promising green innovation activities. Pacheco and Dean [47] found that, in market competition, green technology innovation is related to the future survival and development of enterprises, and that in order to maintain competitive advantages, it is affected by the change signals of the GTI strategies of peer enterprises in the process of digital transformation and has similar imitation reactions. Mubarak et al. [48] argue that the digital transformation of enterprises triggered by the update of industrial information technology can improve the efficiency of information sharing and promote knowledge accumulation, thus, enhancing the green innovation performances of enterprises.

The coupling coordination degree reflects the degree of harmony and consistency between systems, reflecting the change trend from disorder to order after the system interaction [49]. Urban digitalization and green development are both vital drivers and objective functions of high-quality development, and they have developed corresponding development characteristics, modes and concepts in their respective development processes. The combination of digitalization and green development, and the realization of the coordinated drive of the two, are important for economic and social development. However, the current research on their coupling coordination is still in its infancy, with a few scholars preliminarily exploring it from the perspectives of enterprises [50] and digital finance [51].

In summary, we believe that the existing literature can be supplemented and improved from the following perspectives: (1) A systematic review of the synergistic relationship between digitalization and green development. In the existing literature, researchers only discuss the one-way impact of the former on the latter, and few scholars have implemented systematic studies and discussions on the synergistic relationship between the two. (2) A quantitative study on the synergies between digitalization and green development. The existing literature lacks a comprehensive indexing system to build synergies between digitalization and green development. Correspondingly, an in-depth discussion of the spatial–temporal differences and evolutionary characteristics of their codevelopment levels is even more scarce. (3) An assessment of the digitalization and green development at the regional city level. In the existing research on the inter-regional comparisons between digitalization and green development, researchers have mainly focused on the regional and provincial levels, which have complex structures and are not conducive to in-depth analyses of the interaction relations.

## 3. Theoretical Analysis

**Figure 1:**
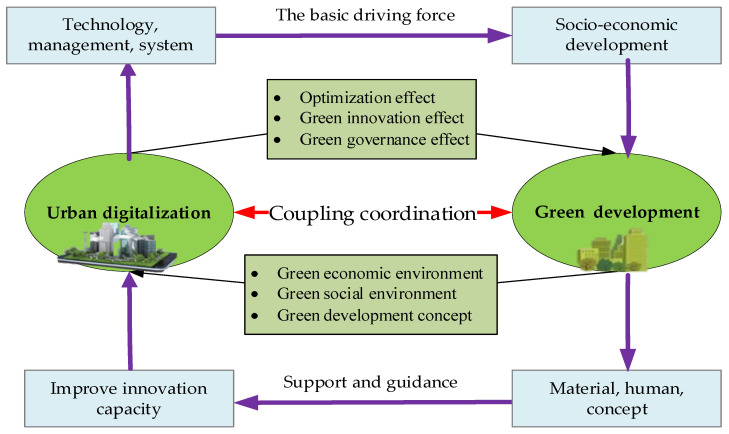
Coupling coordination mechanism of urban digitalization and green development.

We present the theoretical framework for the coupling coordination between urban digitalization and green development in Figure 1. The relationship between urban digitalization and green development is one of mutual promotion, and it is a long-term and dynamic historical evolution. Urban digitalization provides the impetus and guarantees for green development. Urban digitalization promotes economic and social development, transformation and upgrading by promoting technological innovation, management innovation and system upgrading, thus, driving green development. Green development has created a favorable development environment for urban digitalization and sets the current targets for it. Green development can also attract high-quality material and human capital, increasing its popularity, and thus, provide strong support and guidance for urban innovation capacity improvement and further promote urban digitalization.

### 3.1. The Influence of Urban Digitalization on Green Development

Urban digitalization mainly promotes green development in the following three ways: (1) The optimization effect. From a market perspective, urban digitalization improves the efficiency of the production, distribution and consumption in the whole supply chain by providing technical support, data support and algorithm empowerment for traditional industries [52], which makes the industrial hierarchy more reasonable and sustainable and has given rise to new technologies, such as 3D printing, industrial robots and digital twins; these have greatly improved the utilization efficiency and sustainable utilization rates of resources when applied to production and operation [53], replaced and reduced the emissions of harmful gasses, enhanced and improved the recycling of waste and promoted the green development of cities. From a regulatory perspective, the development of digital technology has facilitated the disclosure of industry information and broadened the channels for urban environmental regulation. Digital technology has opened the traditional supervision channel [54] and changed it from the traditional vertical supervision form under the leadership of the government to the multidirectional supervision form under the leadership of the government, masses and society. The public has become one of the main forces for regulation, which has considerably increased the intensity of environmental supervision and strengthened the awareness of corporate environmental responsibility, facilitating the green transformation of cities.

(2) The green innovation effect. On the one hand, technological innovation can improve the levels of intelligent production, resource productivity [55] and pollutant treatment efficiency [56] by accelerating the application and transformation of digital technology in production processes, which promotes the more effective secondary recycling of resources [57] and reduces carbon emissions [58] and additional environmental risks [55], thus, exerting its positive impact on green development. On the other hand, digitalization accelerates information flow, which increases the speed and reduces the cost of knowledge spillover and knowledge interaction in green innovation networks. By relying on digital and information technology, innovation subjects can more conveniently obtain knowledge and information and more quickly master and accumulate emerging knowledge and skills [59]. They can then obtain external information, improve the reserve of innovative knowledge, promote technological upgrading and realize the green transformation of industry and society. In addition, due to the introduction of digital technology, the learning and communication activities among technology departments are more convenient [60], which not only accelerates the generation of green innovation, but also helps the parties involved in supply and demand to better understand the demand for it and the benefit of a reduction in useless innovation.

(3) The green governance effect. Digital governance is the main position of urban digital transformation; it is based on data and takes sharing as the approach, promoting data fusion and realizing cross-level, cross-system, cross-regional and cross-department collaboration, which is a new approach for government and social governance [61]. Data are the most influential factors in production, transforming it into competitiveness, service and creativity. With the help of digital governance means, cities accelerate their industrial agglomeration and inter-regional factor circulation, thus, improving the utilization efficiency of urban resources, forming a circular economic system and reducing pollutant emissions [62], thereby making the urban development greener [63]. Digital governance has broken the spatial barriers to cooperation between cities [64]. Some nonphysical industries can even form cloud industrial clusters [65], which can effectively improve the efficiency of the economic operation and contribute to the green development of cities.

### 3.2. The Influence of Green Development on Urban Digitalization

(1) The green economic environment. The green economy advocates the decoupling of economic growth from ecological consumption to achieve a green and sustainable growth model. The development mode of the green economy proposes the green requirements for the development mode of the digital economy [66]. The scarcity of resources and the requirements of green development are conducive to eliminating traditional industries with elevated levels of energy consumption and pollution, accelerating the innovation of green technology, and promoting the rationalization and optimization of the industrial structure [67], which is an essential driving force for digital technology innovation [68] and an essential test standard for the quality of digital development.

(2) The green social environment. Currently, the principal contradiction facing Chinese society is between the unbalanced and inadequate development and people’s ever-increasing demand for better lives. The requirement of a green society to achieve a harmonious coexistence between humans and nature is an influential driving force for digital construction [69]. At the same time, a beneficial ecological environment and quality of life promote the pursuit of an even higher quality of life. The improvement in people’s quality of life and the awareness of environmental protection can provide a good environment and continuous talent accumulation for the research and development of digital technology [70].

(3) The green development concept. Going green and low carbon is not only in line with the trend of world economic and social development, but it has also become an inherent requirement and inevitable choice for China’s transition to high-quality and sustainable development. Green development has become the primary goal of urban digital development [71]. Urban digital development faces the triangular problem of choosing between “safety, speed and energy consumption”. Under the concept of green development, urban digitalization not only focuses on the price target pursued by the economic system itself, but also considers the natural environment in which the system is located [72], which overcomes the disadvantages of merely incorporating technology into the market and taking price as the guidance.

## 4. Research Design

**Figure 2:**
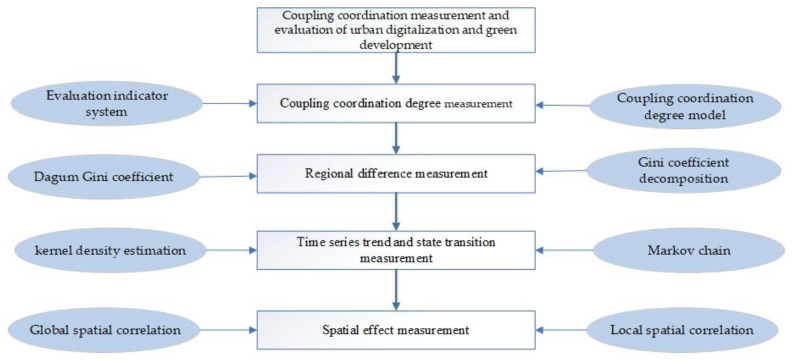
The research framework and the research methods.

Figure 2 shows the overall research framework and methodology design of this paper.

### 4.1. Measurement Method

#### 4.1.1. Coupling Coordination Degree Model

In this paper, we construct a coupling coordination model of urban digitalization and green development to study their synergistic effects. The coupling coordination degree model (CCDM) was widely adopted to explore the coupling relationship and the interaction degree between different systems. CCDM includes two aspects: the degree of coupling and the degree of coordination. The degree of coupling reflects the degree of interrelation between the systems. The higher the degree of coupling, the stronger the correlation between the systems. The degree of coordination represents the degree of mutual promotion between subsystems. The coupling coordination degree combines the coupling and coordination degrees to simultaneously describe the development and coordination levels between the systems. The specific formula of the coupling coordination degree is as follows [73]:

(1) $$CD\&GDC=\sqrt[{2}]{CDL+GDL}/\left(CDL+GDL\right)$$

(2) T=α∗CDL+β∗GDL

(3) $$CD\&GDD=\sqrt[{}]{CD\&GDC*T}$$

(4) E=CDL/GDL

Among them, CDL represents the digitalization level of the city and GDL stands for the green development level; CD&GDC represents the level of coupling between urban digitalization and green development; T is the comprehensive coordination index; CD&GDD is the coupling coordination degree. Considering that urban digitalization and green development are equally important in the process of social development [74], that is α = β = 0.5.

According to the previous research results and the actual situation [75], CD&GDD types are subdivided into low degree (0 < CD&GDD ≤ 0.3), moderate (0.3 < CD&GDD ≤ 0.5), height (0.5 < CD&GDD ≤ 0.8), extreme (0.8 < CD&GDD ≤ 1) coupling coordination of four basic types. E represents the relative development degree of urban digitalization and green development. When 0 < E ≤ 0.8, it means that the digitalization of the city lags behind and restricts the green development; when 0.8 < E ≤ 1.2, it indicates that the two develop synchronously; when E > 1.2, it indicates that green development lags behind and restricts urban digital development.

#### 4.1.2. Regional Difference Measurement Model

In this paper, we use the Dagum Gini coefficient to measure the regional differences of CD&GDD and decompose them. Compared with the difference measurement models such as the Theil index, comprehensive entropy index and coefficient of variation, the Dagum Gini coefficient model further decomposes the dimension of difference into different sources on the basis of measuring the difference, so as to specifically discuss the impact of different groups on the overall difference [76]. Based on the CD&GDD differences calculated from the Dagum Gini coefficient model, we decompose the dimensionality reduction in the differences into three parts: intra-group differences, inter-group differences and hypervariable densities. Within-group differences refer to differences in the interaction effects between prefecture-level cities within a region, while between-group differences refer to differences in the interaction effects between these groups when different groups are analyzed as a whole. We construct the hypervariable density from the overlap between different groups. In other words, not all cities in a certain region have a higher (lower) CD&GDD than cities in another region, which leads to the so-called “high-low” and “low-high” overlapping problems between different regions. The specific calculation process is as follows [76]:

(1) Calculate the overall Gini coefficient *G*:

(5) $$G=\frac{\sum_{\alpha =1}^{l}\sum_{\beta =1}^{l}\sum_{i=1}^{n_{\alpha }}\sum_{j=1}^{n_{\beta }}\left|D_{\alpha \beta }-D_{ij}\right|}{2n^{2}\mu }$$

where $D_{\alpha \beta }$ is the coupling coordination degree of the β city in the α region; n is the number of cities, and l is the number of regions; μ is the average value of CD&GDD in each region.

(2) Calculate the Gini coefficient $G_{\alpha \alpha }$ of the α region and the intra-regional difference $G_{\omega }$. The formula is as follows:

(6) $$G_{\alpha \alpha }=\frac{\sum_{i=1}^{n_{\alpha }}\sum_{j=1}^{n_{\alpha }}\left|D_{\alpha \beta }-D_{ij}\right|}{2n_{\alpha }^{2}\mu_{\alpha }}$$

(7) $$G_{\omega }=\sum_{\alpha =1}^{l}G_{\alpha \alpha }p_{\alpha }s_{\alpha }$$

where $p_{\alpha }=\frac{n_{\alpha }}{n},s_{\alpha }=\frac{n_{\alpha }\mu_{\alpha }}{n\mu }$.

(3) For the Gini coefficient $G_{\alpha \beta }$ between α and β regions and the difference contribution rate $G_{nb}$ between regions, the formula is as follows:

(8) $$G_{\alpha \beta }=\frac{\sum_{i=1}^{n_{\alpha }}\sum_{j=1}^{n_{\beta }}\left|D_{\alpha \beta }-D_{\beta j}\right|}{n_{\alpha }n_{\beta }(\mu_{\alpha }+\mu_{\beta })}$$

(9) $$G_{nb}=\overset{l}{\underset{\alpha =2}{\sum }}\overset{\alpha -1}{\underset{\beta =1}{\sum }}G_{\alpha \beta }\left(p_{\alpha }s_{\beta }+p_{\beta }s_{\alpha }\right)Q_{\alpha \beta }{}_{}$$

(10) $$Q_{\alpha \beta }=\frac{d_{\alpha \beta }-p_{\alpha \beta }}{d_{\alpha \beta }+p_{\alpha \beta }}$$

(11) $$d_{\alpha \beta }=\overset{\infty }{\underset{0}{\int }}dF_{\alpha }\left(y\right)\overset{y}{\underset{0}{\int }}\left(y-x\right)dF_{\beta }\left(x\right)p_{\alpha \beta }\;\;\;\;\;\;\;\;\;\;\;\;\;\;\;\;\;\;\;\;\;\;\;\;\;\;\;\;\;\;\;\;\;\;\;\;\;\;\;=\overset{\infty }{\underset{0}{\int }}dF_{\beta }\left(y\right)\overset{y}{\underset{0}{\int }}\left(y-x\right)dF_{\alpha }\left(y\right)$$

where $Q_{\alpha \beta }$ represents the relative influence of CD&GDD between α and β regions; $d_{\alpha \beta }$ and $p_{\alpha \beta }$ represent the mathematical expectation of the sum of all sample values in the region α and β, which satisfies the condition of $(D_{\alpha \beta }-D_{\beta j})>0$ and $(D_{\beta j}-D_{\alpha \beta })>0$, respectively.

(4) Calculate the supervariable density contribution $G_{l}$, and the formula is as follows:

(12) $$G_{l}=\overset{l}{\underset{\alpha =2}{\sum }}\overset{\alpha -1}{\underset{\beta =1}{\sum }}G_{\alpha \beta }\left(p_{\alpha }s_{\beta }+p_{\beta }s_{\alpha }\right){\left(1-Q_{\alpha \beta }\right)}_{}$$

#### 4.1.3. Time Series Trend Measurement Model

In this paper, we analyzed the trend of the time series evolution of CD&GDD using kernel density estimation. The kernel density method is a nonparametric evaluation method for the study of nonequilibrium distributions. The kernel density estimation method first performs statistics on the probabilities of random variables and then uses continuous probability density curves to show the distribution form of the indicator data, which can better explain the dynamical evolution mechanism of these data. The basic formula of the kernel density function is as follows [77]:

(13) $$f_{N}\left(X\right)=\frac{1}{Nh_{N}}\overset{N}{\underset{i=1}{\sum }}K\left(\frac{X_{i}-\bar{X}}{h_{N}}\right)$$

(14) $$K\left(x\right)=\frac{e^{-0.5x^{2}}}{\sqrt{2\pi }}$$

where $f_{N}\left(X\right)$ is the kernel density estimation function of random variable X; N is the number of cities; K(x) is the kernel function of variable X; $h_{N}$ is the bandwidth, which is a positive number greater than 0, reflects the estimation accuracy and is proportional to the smoothness of the kernel density curve.

#### 4.1.4. State Transition Measure Model

In this paper, we further use a Markov chain to measure the state transition probability of CD&GDD in the next year. The Markov transition matrix is a Markov process with discrete time and state, which reveals the one-step spatial transition probability $m_{ij}$ of a spatial unit belonging to type i in a certain year and transferring to type j in the next year. When the transition reaches stability, then the transition probability of the current year is independent of the initial year. Where the state of a random variable changes over time, this is called a transition. The transition probability of a Markov chain in state i at time t and in state j at time t+1 is:

(15) $$m_{ij}=p(\left(D_{t+1}=j\right)|D_{t}=i)$$

Then, given the observed value of i in the state, the maximum likelihood estimates of the probability of being transferred to state j at time t is given by:

(16) $$m_{ijt}=\frac{n_{ijt}}{n_{it}}$$

By weighting the transition probabilities within the study range of all observed values, the probability of transition from initial state i to state j is obtained as follows [78]:

(17) $$m_{ij}=\frac{\sum_{t=0}^{T}n_{it}m_{ijt}}{\sum_{t=0}^{T}n_{it}}$$

where $n_{i}$ is the number of cities in state i is a given period, and $m_{ijt}$ is the number of cities in state i in year t at a given period and transferred to state j at year t+1.

Before computing the Markov transition matrix, the transition state must first be determined. According to the above calculation results, the CD&GDD of the samples is in the range of (0.2, 0.8). The breakpoint values for the CD&GDD quartiles for the entire sample are 0.3661, 0.4351 and 0.4815, respectively. As a result, in this paper, we divide the relative shift status of CD&GDD into less than 0.3661, 0.3661–0.4351, 0.4351–0.4815 and greater than 0.4815, indicating that the CD&GDD status is low level (I), medium-low level (II), medium-high level (III) and high level, respectively (IV), and then calculate the state transition probability.

#### 4.1.5. Spatial Effect Measurement Model

This paper uses Exploratory Spatial Data Analysis (ESDA) to test the spatial distribution of data and reveal the spatial structure of data and the interaction between subjects [79]. Moran’s I is one of the remarkable methods, including global Moran’s I and local Moran’s I.

(1) Global spatial correlation. The global Moran’s I can be used to determine whether there is a spatial correlation distribution in the data of the subject or phenomenon as a whole. The value of global Moran’s I is [−1, 1]. If the value is closer to 1, the spatial clustering features are more pronounced, and the spatial differences are smaller. If the value is close to −1, the spatial clustering feature is less pronounced, and the spatial difference is larger. CD&GDD may have spatial dependence or autocorrelation at the city level, and global Moran’s I is used to measure the spatial autocorrelation of CD&GDD. The calculation formula is [80]:

(18) $$GMI=\frac{\left(\frac{m}{\gamma_{0}}\right)\times \left\{\sum_{i=1}^{m}\sum_{j=1}^{n}W_{ij}\left(D_{i}-\bar{D}\right)\left(D_{j}-\bar{D}\right)\right\}}{\sum_{i=1}^{m}{\left(D_{i}-\bar{D}\right)}^{2}}$$

where m is the number of prefectural-level cities and n is the number of neighboring cities. $D_{i}$ and $D_{j}$ are the coupling coordination degrees of cities i and j, and $\bar{D}$ is the mean value of the coupling coordination degrees. $\gamma_{0}$ is the element of the standardized weight matrix. $W_{ij}$ is the spatial weight matrix.

(2) Local spatial correlation. The local Moran’s I can determine whether there is an agglomeration effect of subject or phenomenon data in the local space. In order to reflect the atypical characteristics of local states, this paper used the local Moran’s I to investigate the local spatial correlation of CD&GDD, and the calculation formula is as follows [81]:

(19) $$LMI=\frac{D_{i}-\bar{D}}{S^{2}}\sum_{j\neq 1}^{n}W_{ij}\left(D_{j}-\bar{D}\right)$$

where $D_{i}$ and $D_{j}$ are the coupling and coordination degrees of cities i and j, and is the spatial weight matrix. $S^{2}$ is the variance of the coupling coordination degree.

The local Moran’s I can divide the local spatial correlation between the target city and its surrounding cities into four quadrants of spatial agglomeration, and the significance degree is represented by the LISA significance test. The first type is that the level of the city and its surrounding cities is high, referred to as high-high (H-H); the second type is the low level of the city and its surrounding cities, referred to as low-low (L-L); the third category is that the city has a high level and is surrounded by low-level cities, referred to as high-low (H-L); the fourth type is that the city has a low level and is surrounded by a high-level city, which is referred to as low to high (L-H). Within the research space, if the local Moran’s I index is less than 0, it means that the spatial units with different attribute values of “L-H” and “H-L” are clustered together. If the local Moran’s I index is greater than 0, it means that the spatial units with similar attribute values of “H-H” and “L-L” are clustered together [82].

### 4.2. Indicators and Data

We present the specific evaluation indicator system for urban digitalization and green development in Table 1. At present, the digitalization level is mainly measured at the provincial level, and there are two measurement methods, as follows: (1) The single-indicator approach, which uses a specific indicator—such as the digital infrastructure, digital economy application or digital industry development—to construct the index system and calculate the digital economy index [83]; (2) The direct use of the digital financial inclusion index or digital economy index published by the Tencent Research Institute to reflect the digital economic development [84]. Comprehensively considering the various dimensions of urban digitalization and the availability of data, we constructed the urban digitalization indicator from three aspects: (1) the digital foundation; (2) the digital benefit; (3) the digital development.

Green development essentially refers to the mode of achieving high-quality economic and social development under the constraints of the ecological environment. The concepts of green development, sustainable development and China’s ecological civilization are in line, emphasizing the coordination and coupling of the three systems of environment, economy and society [85]. Specifically, the greening of the economy is the core embodiment of green development, the greening of the environment is the carrying basis of green development and the greening of society is the internal support for green development [18]. Starting from the connotation of green development, we drew on the definition of sustainable development by the United Nations [86] and in relevant documents issued by Chinese government departments. We constructed a green development evaluation index containing three first-level indicators (ecology, economy and society) and 14 s-level indicators.

We took the abovementioned variable data from the China Urban Statistical Yearbook (2012–2020), China Regional Statistical Yearbook (2012–2020), and statistical yearbooks and statistical bulletins of all provinces and cities. We used the Digital Financial Inclusion Index from the Index System and Index Compilation of Digital Financial Inclusion published by the Internet Finance Research Center of Peking University [87]. We filled in some of the missing values by linear interpolation and adjacency annual significance. We based the price data in this paper on 2000 prices, and we inflated them. After removing the prefecture-level cities with missing data, we finally obtained a sample of 2538 observations of 282 cities at the prefecture level and above in China from 2011 to 2019. To remove the effect of dimensionality, we applied the range method to normalize the primary data. Then, we measured the CDL and GDL, applying the entropy method [88].

## 5. Results and Analysis

### 5.1. Analysis of Coupling Coordination Degree between Urban Digitalization and Green Development

#### 5.1.1. Full Sample Analysis

**Figure 3:**
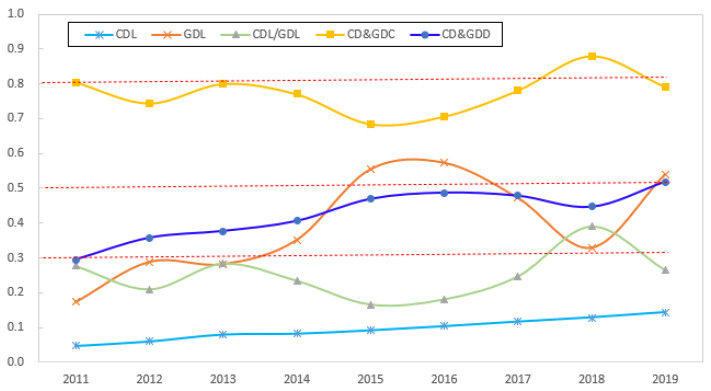
Time characteristics of coupling coordination relationship between urban digitalization and green development from 2011 to 2019.

Figure 3 presents the evolving characteristics of the digitalization and green development levels and their coupling and coordination relations for 282 cities at the prefecture level and above in China from 2011 to 2019. From the perspective of the respective development levels of urban digitalization and green development, the overall level is low but rapidly increasing. China’s CDL and GDL rose from 0.0478 and 0.1727 in 2011, respectively, to 0.1432 and 0.5402 in 2019, respectively, representing increases of 199.5 and 212.7%, respectively. During the sample period, the CDL and GDL were mainly in the range of (0.10, 0.30), with large fluctuations, which indicates that the level of urban digitalization in China is significantly behind the level of green development, which restricts the latter.

From the perspective of the coupling degree, China’s CD&GDC was essentially stable between 0.70 and 0.80 during the sample period, with a relatively concentrated distribution and high coupling stage. The coupling degree reflects the degree of the interaction between the systems, and according to the results, there is a strong and relatively stable correlation between the urban digitalization and green development in the country.

From the perspective of the coupling coordination degree, the CD&GDD in the sample period mostly ranged between 0.30 and 0.50, which is the stage of moderate coupling coordination. No city was in the extreme-coupling-coordination range, and the cities in the high-coupling-coordination range accounted for only 5.32% of the total sample. The cities with moderate coupling coordination accounted for 37.23% of the entire sample, which included 105 cities. A total of 162 cities had low-degree-coupling coordination, accounting for 57.45% of the total sample. The level of coordination between urban digitalization and green development in China remains at a low level, and there is much room for improvement. The CD&GDD shows a significant upward trend over time, with an average annual growth rate of 8.4%. In addition, the value of the coupling coordination degree for each city is typically lower than the value of the coupling degree, which indicates that the coordination between digitalization and green development in Chinese cities is still at a low level of development.

#### 5.1.2. Analysis by Region

**Figure 4:**
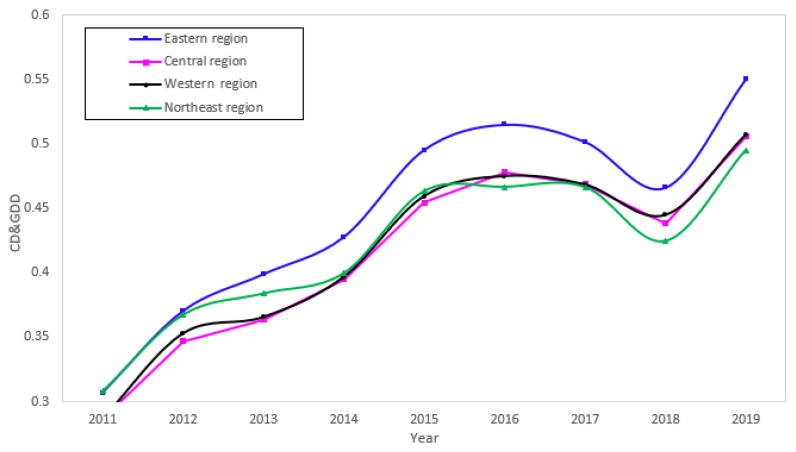
Changes in average values of CD&GDD in China’s four major economic zones from 2011 to 2019.

Figure 4 presents the basic characteristics of the CD&GDD for the four major economic zones in China from 2011 to 2019. Overall, the CD&GDD levels are substantially higher in the eastern regions than in the central, western and northeastern regions, and the CD&GDD levels considerably vary from region to region. Although there is a strong correlation between urban digitalization and green development, a mature and stable interactive development relationship between the two has yet to be established due to the short development time of urban digitalization and the low integration of digitalization with traditional economic and social development. From a time series perspective, the CD&GDD of the four economic zones all show a fluctuating upward trend, which indicates that the benign interplay between digitalization and green development will continue to improve as urban digitalization develops and its broad penetration into the economy and society continues. By region, the CD&GDD in the eastern region is higher than the national average, increasing from 0.3069 in 2011 to 0.5499 in 2019, with an average annual growth rate of 8.8%. The CD&GDD in the central and western regions are extremely close to and slightly below the national average, increasing from 0.2865 and 0.2864 in 2011, respectively, to 0.5060 and 0.5070 in 2019, respectively, with an average annual growth rate of 8.5%. The CD&GDD in northeast China fluctuated considerably, with a mean value higher than those of central and western China from 2011 to 2015, but lower than those of central and western China after 2015. The gap has gradually widened, increasing from 0.3083 in 2011 to 0.4953 in 2019, with an average annual growth rate of 6.74%.

To further analyze the reasons for the large differences in the CD&GDD values between regions, we measured the descriptive statistical measures of the integrated CDL and GDL scores for the four economic zones during the observation period, and we present the results in Table 2. The regional differences were substantially higher in the CDL than in the GDL. The average composite score of the CDL has a maximum of 0.82 and minimum of 0.01, with the maximum being 82 times the minimum. The average GDL composite score has a maximum of 0.93 and minimum of 0.10, with the maximum being 9.3 times the minimum. The huge difference in the urban digitalization levels between the regions is the main reason for the imbalance in the CD&GDD. As the pilot area of urban digital development in China, the eastern region has more mature infrastructure, application channels, mechanisms and conditions for digital development, and it is highly valued by the government, with certain resource endowments and a “siphon effect.” In fact, as early as 2011, the National Development and Reform Commission (NDRC) issued the Notice on the Pilot Work of Carbon Emission Trading, officially approving seven provinces and cities, including Beijing, Tianjin and Shanghai, to launch the pilot work of carbon emission trading. From 2013 to 2014, carbon markets were established in Shanghai, Beijing, Guangdong, Tianjin, Hubei and Chongqing. While the economic foundations of the central and western regions are relatively weak compared with the eastern regions, the marginal effect of their development is more substantial due to the strong support of policies, such as the development of the western regions and the rise of the central regions. Of course, there is still a substantial gap between the CD&GDD in the central and western regions and the CD&GDD in the east. The economic structure of northeast China lags behind, with a relatively high proportion of traditional industries, numerous constraints on green development and a low level of digital infrastructure, which may result in a low CD&GDD. Reducing the imbalances in regional development, and especially in urban digital development, should be the main direction of related policies in the future.

### 5.2. Analysis of Regional Differences in CD&GDD

#### 5.2.1. Overall Differences

In this study, we calculated the Gini coefficients of the CD&GDD in China from 2011 to 2019 with Matlab2019 software (Mathworks: Natick, MA, USA), based on the method of the Dagum Gini coefficient calculation. We present the overall Gini coefficients and their decompositions for the CD&GDD from 2011 to 2019 in Table 3. The overall CD&GDD distribution in China showed a downward trend in volatility during the observation period, with a total decrease of 23.49%. The annual average of the overall Gini coefficient for the four economic zones was 0.0548 during the observation period. In terms of time, the Gini coefficient decreased from 0.0674 in 2011 to a minimum of 0.0451 in 2016, and then slightly increased to 0.0545 in 2019, which indicates that, with the implementation of China’s coordinated regional development policies, such as the modernization of the eastern region, development of the western region and rapid rise of the central region [89], the regional imbalance between the urban digitalization and green development levels has been alleviated. Although the Gini coefficient declined overall, its absolute value remains large and shows signs of rebounding, which indicates that the problem of the unbalanced development of the CD&GDD in different regions in China is still substantial, and that reducing the problem of unbalanced regional development should be the direction of relevant policies in the future.

In terms of the differential contributions, for all but 2015, 2016 and 2019, the rates of contribution to the overall Gini coefficient ranged from large to small in terms of the hypervariable density, net inter-regional differences and intra-regional differences. To some extent, the above phenomena indicate that the hypervariable density and inter-regional differences are the main sources of the overall differences. In terms of time, the intra-regional differential contribution is mainly stable in each year, with no large fluctuations and a small contribution rate, and with an average annual contribution of 26.18%, which means that the corresponding policy effect of the state to reduce the intra-regional differences has been effectively implemented, and the division of the major strategic regions is justified but still needs to be maintained. The contribution rate of the hypervariable density shows a fluctuating and decreasing trend, which contrasts with the trend of the contribution rate of the net difference between the regions. The hypervariable density is the contribution to the overall gap that is caused by the existence of the cross term when dividing molecular groups, and researchers use it to identify the cross-overlap phenomenon between regions [90]. Thus, according to the results, the early inter-regional differences were mainly caused by the extremely high CD&GDD of some cities in some regions, and by the extremely low CD&GDD of some cities in other regions, which indicates some polarization. However, in recent years, this polarization trend has been relatively weakened, and the inter-regional differences consist mainly of the net differences between the regions.

#### 5.2.2. Differences within Four Regions

**Figure 5:**
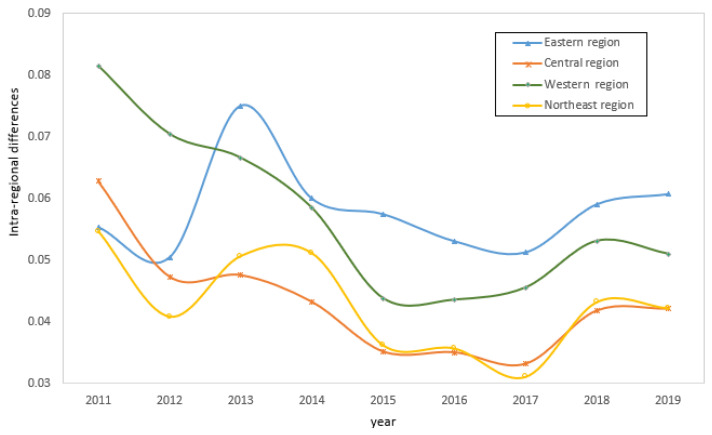
Gini coefficient of CD&GDD within four economic zones from 2011 to 2019.

Figure 5 presents the CD&GDD Gini coefficients for the four economic zones from 2011 to 2019. During the observation period, the mean values of the CD&GDD Gini coefficients for the four economic zones showed an overall decrease, which was accompanied by some fluctuations. The intra-regional differences in the CD&GDD levels were the largest but were relatively stable in the eastern region, which was followed by the western region, which had the largest range of fluctuations. The intra-regional differences in the CD&GDD levels were not high in the central and northeastern regions, but both need to be further stabilized. Regionally, the intra-regional Gini coefficient in the eastern region expanded rapidly before 2014, then decreased and tended to stabilize, which indicates that the CD&GDD difference in the eastern region became increasingly large before 2014 and had a converging trend after that. Prior to 2016, the intra-regional difference in the west showed a sharp downward trend, falling from 0.0805 in 2011 to 0.0435 in 2016, but it has significantly recovered since 2017. The intra-regional differences between the central and northeastern regions were relatively close and fluctuated continuously, but there was a downward trend overall. The above results once again confirm the problem of the imbalanced development within each region at the CD&GDD level in China, and the need to combine the development characteristics of each region in the formulation of coordinated regional development policies.

#### 5.2.3. Differences among Four Regions

**Figure 6:**
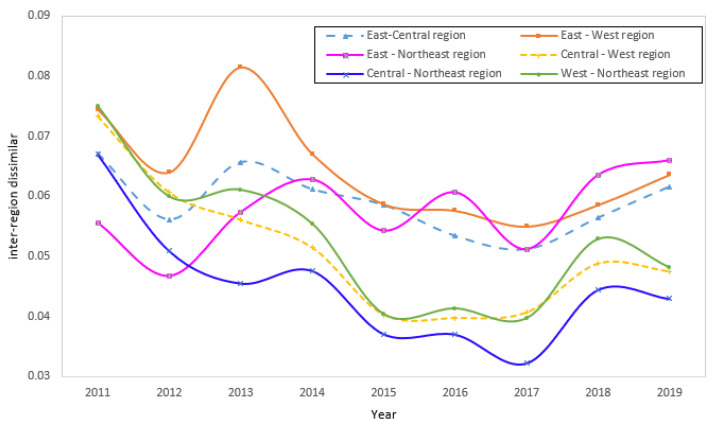
Gini coefficients of CD&GDD among four major economic zones from 2011 to 2019.

Figure 6 presents the CD&GDD Gini coefficients between the four major economic zones from 2011 to 2019. From a time-trend perspective, the CD&GDD Gini coefficient was in a state of continuous fluctuation between the regions, but it showed an overall downward trend. The central and northeastern regions experienced the largest reduction in regional variation, with a decrease of 56%. The eastern and central regions showed the smallest decrease in regional variation, with a decrease of 8.83%. The difference between the central–western and western–northeast regions also decreased by more than 50%. The east–northeast regional Gini coefficient showed an expanding fluctuation trend, which indicates that, in the east and northeast cities, the digitization and the development of the green policy enforcement and their effects are not consistent and need additional promotion, according to the features of the development of the two regions, to develop more targeted policies and measures to narrow the CD&GDD differences between the regions. During the observation period, the difference between the eastern region and other regions was substantially higher than the differences between the other regions. The east–west group had the largest difference, with an annual average difference of 0.0645. The central and northeastern regions had the smallest difference between the groups, with an annual average difference of 0.0450. The differences between the other four groups of regions were all above 0.05. According to the above information, the level of the CD&GDD considerably varied from region to region, which is in agreement with previous analyses.

### 5.3. Time Series Evolution Analysis of CD&GDD

#### 5.3.1. Time Evolution Trend Analysis

**Figure 7:**
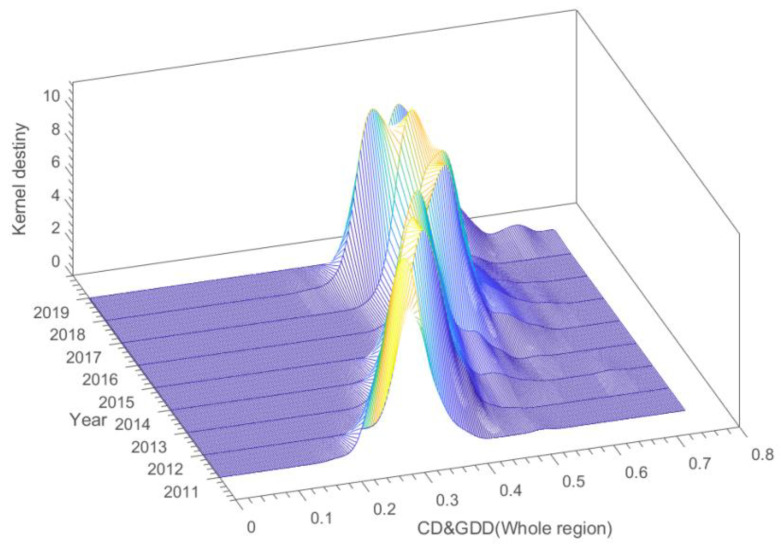
Three-dimensional kernel density map of China’s CD&GDD.

Figure 7 presents a 3D kernel density map of the full CD&GDD sample from 2011 to 2019. For the whole sample, the main peak of the kernel density curve is distributed on the right side of the curve, which indicates that most Chinese cities have high CD&GDD levels. At the same time, the CD&GDD distribution shows a trend to the right, which indicates that the CD&GDD levels in most Chinese cities are on an upward track, and that urban digitalization and green development have been effectively coordinated overall. The shape of the nuclear density curve is relatively stable; the width of the peak also varies with the fluctuation in the peak and there are no clear peaks, troughs or single or double peaks, nor is there a clear polarization trend. The Chinese government regularly attaches great importance to the development of green and digital construction. In 2017, it formally implemented the acceleration of the development strategy of a digital China and Chinese construction wisdom, and local governments are increasingly paying attention to coordination with the development of green city digitalization and the effective integration of the central and local governments to promote, from city to city, the overall increase in the digitalization construction and green development level. The regional difference and polarization trend have been effectively alleviated, and the degree of spatial equalization has been substantially improved. However, given the differences in the resource endowments and environmental constraints in the different regions, the gap between the coupling coordination development of the two regions still exists, which should be continuously noted.

**Figure 8:**
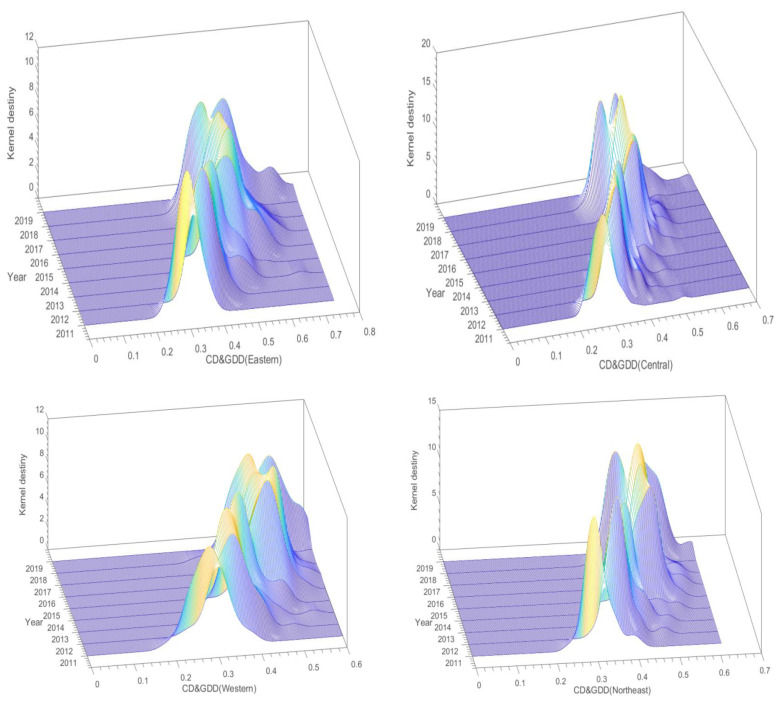
Three-dimensional kernel density maps of CD&GDD in four economic zones.

Figure 8 presents the CD&GDD 3D kernel density maps for the four economic zones. Regionally, the main peak in the eastern region shifts to the right overall, with a well-defined single-peak trend. The peak of the main peak has a “U” shape, which indicates a “decreasing-rising” trend, which is accompanied by a decrease in the width of the main peak, indicating that the CD&GDD in this region has maintained a steady upward trend. According to this performance, the eastern region with a relatively developed economy and strong resource endowment advantages has a “siphon effect,” which means that it can attract capital and talent from across the country. The main peak in the central region moves to the right overall, and a single-peak trend is evident. The height of the main peak has continued to increase, but it has shifted repeatedly from side to side in recent years and is not extremely stable, which indicates that national policies to support the digitalization and green development of the cities in the central region have been effectively implemented and the polarization reduced; however, this needs to be further strengthened and stabilized. The main peak in the western region shifts to the right year after year, which indicates faster CD&GDD growth. However, in recent years, there has been some fluctuation in the peak of the main peak, with a substantial increase in the width, which indicates that the CD&GDD difference in this region is expanding. This is likely due to the fact that digital development in Guizhou is a sudden phenomenon, with the growth rate of the digital economy ranking first in China for seven consecutive years, which has further exacerbated the imbalance between digitalization and green development in the western Chinese cities. In northeast China, the main peak substantially shifted to the right before 2016, and it has remained relatively stable since then. The single-peak trend is evident, but the peak variation is not particularly pronounced, and there is almost no large trend, which indicates that the CDRDD in northeast China is in a relatively stable state after a period of growth, and that the region has entered a bottleneck period for urban digitalization and green development. In terms of the distributional scalability, there are no substantial tails in the kernel density curves for any region, which indicates that the CD&GDD of each region was relatively concentrated in each year, and there is no clear polarization trend.

#### 5.3.2. State Transition Analysis

In this study, we used the Markov transition matrix to reveal the specific relative transition rules of the CD&GDDs in each region. Table 4 presents the results of the CD&GDD Markov transition probability matrix calculations from 2011 to 2019. The rows in the table represent the CDGDD state in year t, and the columns represent the CD&GDD state in year t + 1. The diagonal numbers represent the probability that the CD&GDD state does not change in year t + 1, while the off-diagonal numbers represent the probability that the CD&GDD state changes in year t + 1.

From the perspective of the entire sample, 55.59% of the cities that were in state I in year t remained in state I the following year, while 42.83 and 1.57% of the cities rose to states II and III, respectively, in year t + 1. Of the cities in state II in year t, 36.51% will remain in state II in year t + 1, while 1.59, 43.97 and 17.94% will drop one level, increase one level and increase two levels, respectively, in year t + 1. Of the cities in state III in year t, 42.98% will remain in state III in year t + 1, while 20.53 and 36.49% will decrease one level and increase one level, respectively, in year t + 1. Of the cities in state IV in year t, 74.35% will not switch their status in year t + 1, while 24.23 and 1.43% will drop one grade and two grades, respectively, in year t + 1. Most of the diagonal elements have larger values than the off-diagonal elements, which indicates that the mobility between the CD&GDD states is low, and the stability of the original state is maintained. Among them, the states at both ends of the diagonal (I and IV) have the greatest stability, which reflects, to a certain extent, the possibility of CD&GDD convergence to low and high levels (i.e., the phenomenon of “club convergence”). At the same time, in the off-diagonal state transition, the value on the right side of the main diagonal is typically greater than the value on the left side (i.e., the probability of the upward transition of the coupling coordination state is greater than the probability of the downward transition). This indicates that the CD&GDD has a long-term growth trend, which is consistent with the previous time series analysis results. Moreover, the transition probabilities between the CD&GDDs in the neighboring states are larger than those in the non-neighboring states, which indicates that the state evolution of the CD&GDDs is a relatively continuous and gradual process.

We present the results from the perspectives of the different regions from 2011 to 2019. In the eastern region, the cities in states I and III in year t have relatively high probabilities of moving up in year t + 1. The cities in states II and IV in year t are more likely to remain in these states in year t + 1. Half of the diagonal elements are smaller than the off-diagonal elements, and the transition probabilities between the adjacent states are larger than those between the nonadjacent states. The likelihood of the CD&GDD remaining stable in the eastern region is weak, with an upward trend. In the central region, the cities in states I, III and IV in year t have a greater probability of remaining unchanged in year t + 1. The cities in state II in year t have an upward shift in year t + 1. With the exception of the cities in state II, the diagonal elements are larger than the off-diagonal elements, and the CD&GDD in the central region is more likely to remain stable. In the western region, the cities in states I and IV in year t are more likely to remain in these states in year t + 1. A city in state II in year t has a relatively high probability of moving up one level in year t + 1. However, a city in state III in year t has an equal probability of moving up one level and staying at the same level in year t + 1. In the northeast region, the cities in states I and II in year t are prone to move to the next state in year t + 1. The cities in states III and IV at year t will remain in these states in year t + 1, with a high probability. The possibility of the CD&GDD remaining stable in northeast China is weak, and the high-level state indicates a downward-shift trend.

### 5.4. Spatial Effect Analysis of CD&GDD

#### 5.4.1. Analysis of Global Spatial Effects

##### Overall Spatial Effect

We successively present the measurements of the global spatial correlation of the CD&GDD in China under the two weight matrices of geographical distance (weight W1) and economic distance (weight W2) in Table 5. During the observation period, the global Moran index of the CD&GDD at the national level for both weights was significantly positive at the 1% level, which indicates that the CD&GDD was spatially correlated. Under the geographical-distance-weighting matrix (W1), the Moran index of the CD&GDD showed a fluctuating upward trend, increasing from 0.053 in 2011 to 0.079 in 2019, with an average annual growth rate of 5.45%, which indicates a gradual increase in the spatial correlation of the CD&GDD for cities that are geographically close to each other. Under the weight matrix of economic distance (W2), the spatial Moran’s I of each year passes the significance test at the level of 1%, which indicates that the spatial correlation of the CD&GDD is also characterized by the spatial correlation of the simple differences in the economic development. From the perspective of the evolution trend, the Moran index showed a fluctuating upward trend before 2017, but it has shown a substantial decline in the past two years, indicating that the spatial spillover effect of the CD&GDD in cities with similar economic development has weakened, which has a negative impact on the improvement in the CD&GDD. The possible reason is that China has a vast territory and large differences in resource endowments, industrial structures and ecological environment statuses among cities, which has created the differentiated features of the CD&GDD development path. The “race to the bottom” and “free rider” effects of technological innovation, as well as the objective existence of the transfer and undertaking of “black” industries in the process of green development, also weaken the positive impact of a city on the spatially related cities to a certain extent, thus, causing considerable fluctuation in the spatial spillover effect.

##### Spatial Effects of Four Regions

We present the global spatial Moran’s I values of the CD&GDD for the four economic regions in Table 6. Overall, the Moran’s I was significantly positive only in the eastern and central regions, and it was either positive or negative in the rest of the regions; however, there are relatively few years in which the index was significant. The absolute value of the Moran index shows a fluctuating upward trend in the eastern region, from 0.102 in 2011 to 0.129 in 2019, which indicates that the spatial correlation of the CD&GDD in the eastern region is gradually increasing. The Moran’s I in the central region shows an overall downward trend, and while the significance level remains the same, the value becomes smaller, which indicates that the spatial correlation of the CD&GDD in the central region is decreasing. The western region had a fluctuating downward trend throughout the observation period. The fluctuations in the northeast eventually leveled off.

In terms of correlation, the CD&GDD in the eastern and central regions had significant positive spatial autocorrelation, which indicates that the cities with high CD&GDD in this region are close to each other and exhibit some clustering. In the western region, the spatial autocorrelation was significantly positive until 2017, but it has become negative in the past two years, which indicates that the cities with high CD&GDD in this region have shown an expansion of points in recent years and are far apart. The frequent jumps in the spatial autocorrelation type of the CD&GDD in northeast China indicate that the CD&GDD in this region is less stable.

#### 5.4.2. Analysis of Local Spatial Effects

**Figure 9:**
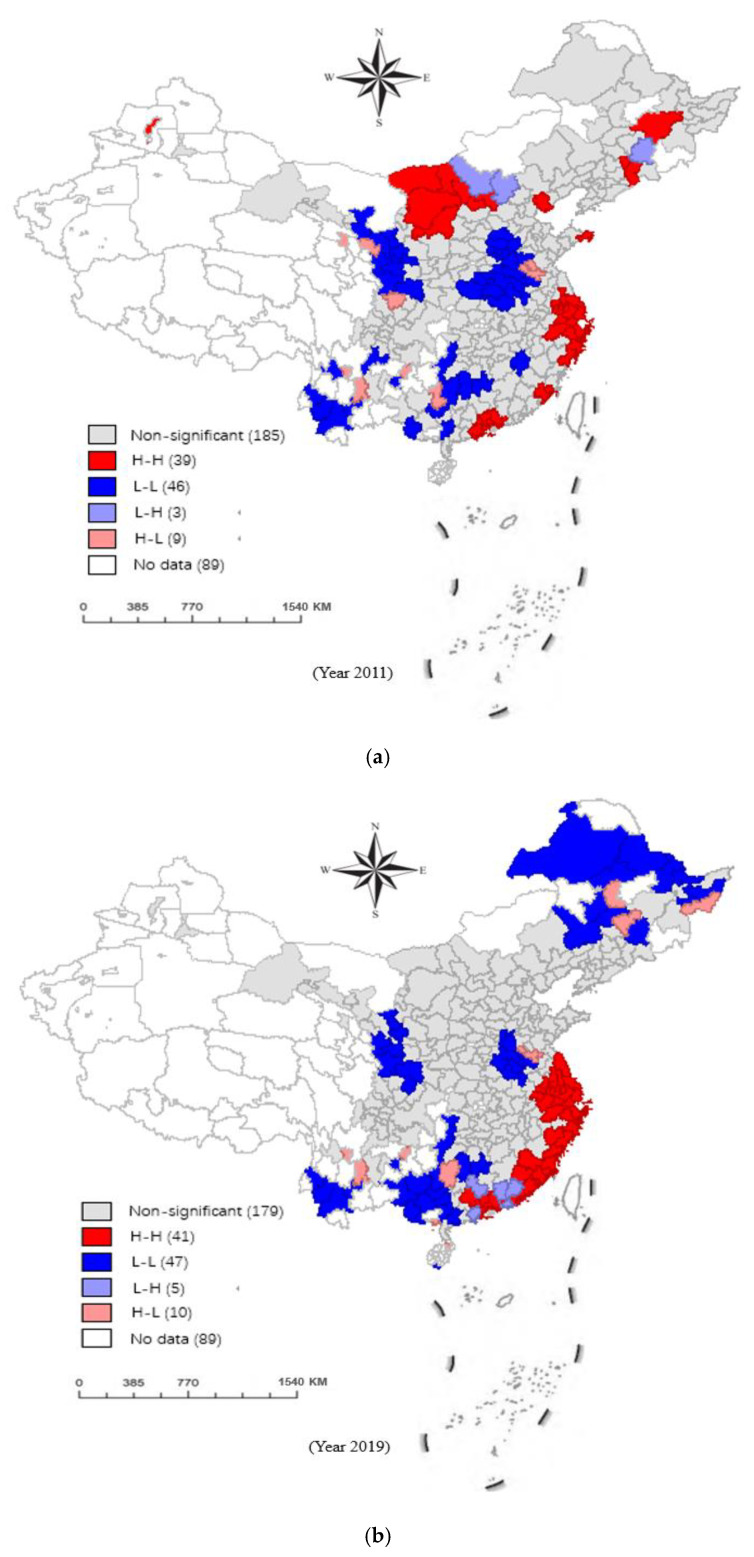
LISA plot of local Moran’s I for CD&GDD in 2011 (**a**) and 2019 (**b**).

We could only use global spatial autocorrelation to analyze the spatial clustering properties of the CD&GDD at the population level, and this does not reflect the spatial pattern differences in the inner regions. Therefore, we used GeoDa1.6 software to calculate the local Moran’s I values in 2011 and 2019, and to draw a LISA agglomeration map (Figure 9).

In general, over the years, the “H-H” agglomeration areas of the CD&GDD have mainly been concentrated in the eastern region, and the “L-L” agglomeration areas have mainly been concentrated in the central and western regions, which indicates the increasing trend of the “H-H” agglomeration areas. Specifically, according to Figure 9a, in 2011, some cities in the central and western regions had L-L agglomeration, and Heze, Hanzhong, Haidong and Liuzhou had H-L agglomeration, which indicates that the areas with low CD&GDD levels in the above regions inhibit the CD&GDD levels in neighboring areas. Some cities in the eastern, central and northeastern regions had a “H−H” type of agglomeration, which indicates that the higher CD&GDD levels in their neighboring regions will promote the CD&GDD levels in their own regions. The “low-high” agglomeration is mainly located in Ulanqab and Zhangjiakou, and the higher CD&GDD in the surrounding areas will inhibit the coordination level in this region.

According to Figure 9b, the local spatial correlation distribution of the CD&GDD level in 2019 substantially changed compared with that in 2011, and the areas with substantial spatial correlation also increased. The “H-H”-agglomeration cities are all in the eastern region and are mainly concentrated in the southeast coastal area. The digitalization and green development of the adjacent cities in the southeast coastal areas have formed a coupling development linkage area with complementary advantages, and the “spatial agglomeration effect” and “spatial spillover effect” of digitalization and green development are obvious. The number of “L-L”-type clusters is still large, while the number of “H-L”-type clusters is small, and they are mainly concentrated in the central, western and northeastern regions, which indicates that the CD&GDD of the surrounding cities will inhibit the coordination level in the region.

According to the above analysis, for regions with relatively high levels of economic development, the CD&GDD of the surrounding region is generally favorable to the coordination level of the region, which may be due to the siphon effect generated by the central city. In a region with a relatively low level of economic development, the CDGDD of the surrounding area will inhibit the coordination level of the region because the siphon effect of the adjacent area reduces the agglomeration of the resources in the region, which is not conducive to the improvement in the CD&GDD level.

## 6. Discussion

In this study, we constructed a coupling coordination index system for urban digitalization and green development based on 2011 to 2019 panel data from 282 cities at the prefecture level and above in China, and we calculated the CD&GDD. We then further measured and decomposed the differences between the CD&GDD in China and the four economic zones using the Dagum Gini coefficient method, analyzed the evolution of the CD&GDD from a time perspective using kernel density estimation and studied the transition probabilities of each state of the CD&GDD by means of the Markov chain. Finally, we employed Moran’s I to analyze the global and local spatial effects of the CD&GDD. We found that there is a strong interplay between urban digitalization and green development in China, and the level of the CD&GDD is continuously improving.

Previous studies on green development have focused on the analysis of the relationship or coordination between energy, environment and economic development [91,92], based on different perspectives such as consumption [93], energy [94], international trade [95] and economic uncertainty [96]. The similarity between this paper and previous studies is that this study also focuses on the unification and coordination of economic development and ecological environment. Unlike previous studies, this paper deals with a wider range of green development, focusing on the unification and coordination of economic development, social progress and ecological construction. From the perspective of the interaction between economy, society and environment, we specifically analyzed the relationship and coordination between urban digitalization and green development.

Systematic research on the spatial–temporal coupling between urban digitalization and green development is essential for optimizing the allocation of data resources to achieve green and sustainable urban development, and it can serve as a reference for urban green development strategies in the context of the digital era. In this study, we comprehensively used various coupled and spatial–temporal effect models to measure and analyze the coupling between urban digitalization and green development in China and the four economic zones, which provides an empirical basis for green sustainable development in China’s urban digitalization construction process. Compared with the existing studies that separately measure urban digitization [97,98] and green development [99,100], or analyze the relationship between the two from the perspective of the unidirectional impact of the former on the latter [12,101], based on the perspective of coordination and interaction, in this study, we systematically measured the spatial–temporal differentiation, dynamic evolution and spatial effects of urban digitalization and green development, analyzed the current pattern and future trend of the CD&GDD, and comprehensively and systematically grasped the development law of the coupling coordination between digitalization and green development at the regional city level to solve the dilemma of the “energy rebound effect” of digitalization [9,102], and to provide a reasonable reference for the realization of urban green and high-quality development.

China’s CD&GDD and the four major economic zones have their own distinct development laws. In this study, we found that the level of the digitalization and green development of Chinese cities showed an obvious upward trend, which is consistent with the studies of existing scholars [62,63]. In contrast to previous studies, we expanded on the features and rules of the coupling coordination relations between the two and discovered fresh results. Overall, the level of urban digitalization in China lags behind the level of green development. The irrational utilization and allocation of regional digital resources has created numerous problems; these are mainly due to the late start of urban digital development in China [103] and have led to urban green development falling behind. In addition, due to natural endowments, the economic foundation and other reasons, there are not only great differences in the levels of economic and social development among the different regions in China, but also unbalanced and inadequate phenomena in digital technology, digital talent and other resources [104]. Inland areas commonly lag behind coastal areas in terms of digital infrastructure, digital administrative services and digital life services [105], which has resulted in obvious spatial differences in the CD&GDD. By region, most of the cities in the eastern region belong to regions with highly coupled and coordinated urban digitalization and green development. With the exception of some growth poles or core regions with good coordination, the regions with low coordination are mainly located in the central, western and northeastern regions of China. Some of these areas lack the necessary foundation for development, which has resulted in low levels of digital technology, which have thus affected the green development [106]. The CD&GDD differences in the four economic zones show large fluctuations, and in recent years, the net inter-regional differences in the CD&GDD have been the main source of the inter-regional differences. Digitalization has penetrated into all aspects of economic and social development [107]. At the same time, according to the results of this paper, the CD&GDD also has a clear spatial spillover effect; thus, it is important to emphasize regional cooperation to facilitate the collaborative development of urban digitalization and greening.

## 7. Policy Implications

The research results of this paper have the following policy implications:

(1) We need to pay attention to the balance between urban digitalization and green development and reduce the polarization phenomenon in various regions. The large regional variation in the CD&GDD levels between the eastern, central, western and northeastern regions is related to the imbalance between urban digitalization and green development in all regions of China. We must not only consolidate the existing development achievements and advantages of the eastern region, but also take care to prevent polarization in some cities. At the same time, we should continue to increase policy support for the central, western and northeast regions. We should pay attention to the speed of improving the level of coordinated development between the regions, continuously strengthen the awareness of the coordinated development between the regions and space, and prevent the “siphon effect”. At the same time, we should accurately understand the imbalance in the synergy between urban digitalization and green development. Balanced development is the goal, while unbalanced development is the norm; a fully balanced development of the two is not realistic.
(2) We need to explore relevant policies to promote the synergy between urban digitalization and green development according to local conditions. The state should thoroughly understand the current situation and characteristics of the coordination between urban digitalization and green development in various regions, and it should formulate appropriate development strategies by comprehensively considering the differences in their economic and social development levels, historical foundations, resource endowments, location conditions, etc. Moreover, we need to identify the core factors to promote the coordinated development level of the CD&GDD in each region, and to further improve the coupling and coupling coordination degrees of urban digitalization and green development. In the eastern region, where the economy, science and technology are more advanced, we should focus on environmental regulation, strengthen the publicity of green concepts and advocate green and low-carbon behavior. In the central and western regions, where green resources are more abundant, the government should increase economic construction, increase investment in scientific research, vigorously develop digital- and green-development-related infrastructure and build a well-developed transportation network. Northeast China, which is not optimistic on either front, should still make the development of the green economy the main theme, enhance its supporting role in advanced productive forces—such as digital technology—and accelerate the pace of green transformation through digitalization.
(3) We need to give full consideration to the global and local positive spatial correlation of the CD&GDD and optimize the spatial correlation pattern of CD&GDD development. We should firmly establish the concept of the new development, encourage neighboring cities to coordinate comprehensive plans for CD&GDD development and promote the formation of a new coordinated development pattern among cities. The central city should fully embrace the role of a synergistic driving force for the surrounding cities, give full consideration to the spatial spillover effect of the synergistic development of the two cities, strengthen the radiating power to the surrounding areas and realize the scope of its influence from the point to the line, and then to the surface. On the premise of maintaining a favorable momentum of development, the cities in the southeast coastal areas and other “H-H” spatial clusters should attach importance to the development of digitalization and green innovation, explore new forms of business and new models and paths of the coupling development of the two, and improve their coordinated development level. At the same time, we should actively give full play to the existing advantages of capital, technology, markets and management, and play a radiating and driving role in neighboring and distant cities through industrial cultivation, resource development and talent training. Cities with “L-L” spatial agglomeration forms, such as those in central, western and northeast China, as well as those with “H-L” and “L-H” spatial agglomeration forms, should adhere to the combination of “internal strength” and “external aid,” deeply explore the internal development characteristics of the city and pay attention to the improvement in the coupling development capacity of the two. At the same time, it is necessary to actively learn the advanced experiences and development modes of cities with “H-H” spatial agglomeration forms, actively integrate them into the comprehensive CD&GDD development planning of such cities, attach importance to benefit sharing and compensation, seek common interest points and establish a coordinated development mechanism of a regional CD&GDD as a link.

## 8. Conclusions

In this study, we analyzed the coupling coordination between urban digitalization and green development, and its dynamical evolution features, from the comprehensive perspective of the time passage, spatial evolution and geographical proximity, and we revealed the spatial dependence of the CD&GDD. This research provides useful policy guidance for China and other similar regions around the world for the formulation of differentiated urban digital development policies and green development strategies. The conclusions of the study are as follows:

First, from the perspective of coupling coordination development, during the sample period, both the urban digitalization and green development in China achieved rapid development; however, the overall level was low, and the level of urban digitalization was substantially behind the level of green development, which has limited green development. The level of urban digitalization and green development in China has not reached a sound state of development, and there is a strong interaction relationship between the two systems. The CD&GDDs of the four economic zones fluctuate with time, but there is a large difference in the levels between the regions, which show a gradually decreasing spatial pattern of “east-central west-northeast”. Huge differences in the urban digitalization levels between the regions are the main reason for the imbalance in the CD&GDD.

Second, there are still large regional differences in China’s CD&GDD levels; however, these are showing an overall downward fluctuating trend. The difference between the regions is its main source, and the CD&GDD difference between the regions is mainly composed of the hypervariable density and net difference between the regions. Other than 2015, 2016 and 2019, the rates of the contribution to the overall Gini coefficient from large to small are hypervariable density, net inter-regional difference and intra-regional difference. The combined contribution rate of the hypervariable density and net inter-regional difference is always above 70%, which indicates that the difference in the CD&GDD between the regions is caused by the extremely high CD&GDD of some cities in some regions, and the extremely low CD&GDD of some cities in other regions, as well as by the net inter-regional difference. Within the range of the study, the central and northeastern regions had the greatest decrease in regional differences, while the eastern and northeastern regions had a trend of increasing fluctuations.

Third, from the perspective of dynamic evolution, China’s CD&GDD level is on an upward track, and urban digitalization and green development have been effectively coordinated. From the regional distribution point of view, the main peak in the eastern region moves to the right overall, with a clear unimodal trend. The height of the main peak in the central region has been increasing, but in recent years, it has frequently shifted from side to side and has not been particularly stable. The main peak in the western region has a clear trend of moving to the right year by year, but in recent years, there has been some fluctuation in the peak of the main peak, while the width has substantially increased, and the difference in the CD&GDD levels in this region is expanding. In northeast China, the main peak substantially shifted to the right before 2016 and remained relatively stable after 2016. From the point of view of the region scalability, the absence of substantial tails in the core density curves of each region indicates that the CD&GDD of each region is relatively concentrated in each year, and that there is no clear polarization trend. From the point of view of the trend of the state transition, it is relatively likely that the CD&GDD level remains stable as a whole and increases step by step. Each region has a varying degree of upward or downward translation.

Fourth, from the perspective of the spatial effects, the CD&GDD in China normally show a high degree of spatial correlation, with some cities having significant “H-H” or “L-L” clustering features. During the observation period, there was a significant spatial correlation between the CD&GDD of cities with close geographical and economic distances, and the spatial correlation of the cities with close geographical distances tended to gradually increase. The CD&GDD in the eastern and central regions had substantial positive spatial autocorrelation, while the coupling coordination in the other regions did not have any substantial spatial correlation in some years. The “high-high” CD&GDD agglomeration areas are mainly concentrated in the eastern region, and they have gradually shifted to the southeast coast in recent years, which indicates an increasing trend. The areas with “low-low” CD&GDD agglomeration are mainly concentrated in the central and western regions, and the number is relatively stable.

There are some limitations to this study. The coupling coordination of urban digitalization and green development is the result of the joint action of multiple factors, which are dynamic and changeable. Therefore, the optimization of the evaluation metrics and the integration of other relevant factors are important issues to be addressed in further research. In addition, this paper only studies the spatial–temporal differentiation, dynamic evolution and spatial correlation pattern of the CD&GDD from the technical level, as well as the influencing factors on the CD&GDD. The microscopic mechanisms of the interactions and couplings among the various elements have yet to be revealed, which is a direction for further consideration and discussion in future studies.

## Figures and Tables

**Table 1:** Indicator system of urban digitalization and green development.

Level One Indicators	The Secondary Indicators	Level 3 Indicators	Indicator Explanation	Indicator Direction
Green development	Green economy	GDP growth rate (%)	−	+
		Percentage of tertiary production (%)	Tertiary industry added value/GDP	+
		Average profit of industrial enterprises (CNY 10k)	Profits of industrial enterprises/Number of industrial enterprises	+
		GDP created per unit of energy consumption (CNY)	GDP/per unit of energy consumption	+
	Green society	Natural population growth rate (%)	−	−
		Number of doctors per 10,000 (people)	Total number of doctors/population at year-end	+
		Public finance spending as a percentage of GDP (%)	Annual public expenditure/GDP	+
		Average salary of employees on the job (CNY)	−	+
		Registered urban unemployment rate (%)	−	+
	Green ecological	Industrial wastewater discharge intensity of CNY 10k GDP (ton/CNY 10k)	Industrial wastewater discharge/GDP	−
		Industrial soot emission intensity of CNY 10k GDP (ton/CNY 10k)	Industrial soot emission/GDP	−
		SO_2_ emission intensity of CNY 10k GDP (ton/CNY 10k)	SO_2_ emissions/GDP	−
		Green coverage rate of municipal district (%)	−	+
		Harmless disposal rate of household garbage (%)	−	+
Urban digitalization	Digital Basics	Internet users per 100 people (households)	Internet users/total population	+
		Computer services and software employees (%)	Total number of employees in computer services and software/Total number of employees	+
	Digital benefits	Total telecom business per capita (CNY)	Total telecommunications business/total population	+
		Postal services per capita (CNY)	Total postal business/total population	+
	Digital development	Mobile phone users per 100 people (households)	Mobile phone users/total population	+
		Digital Financial Inclusion index	Comprehensive	+

**Table 2:** Descriptive statistics of CDL and GDL composite scores for four economic zones.

Regions	CDL			GDL		
	Average	Min	Max	Average	Min	Max
Eastern region	0.121785	0.03	0.82	0.383949	0.1	0.73
Central region	0.081911	0.02	0.43	0.400889	0.11	0.87
Western region	0.082182	0.01	0.25	0.415032	0.1	0.93
Northeast region	0.088642	0.03	0.22	0.378472	0.13	0.85

**Table 3:** Sources and contributions of overall differences in China’s CD&GDD from 2011 to 2019.

Year	Overall	Intra-Regional Differences		Inter-Regional Differences		Hypervariable Density	
		Sources	Contribution (%)	Sources	Contribution (%)	Sources	Contribution (%)
2011	0.0674	0.0178	26.43	0.0174	25.8	0.0322	47.77
2012	0.0557	0.0147	26.36	0.0153	27.51	0.0257	46.13
2013	0.0650	0.0171	26.31	0.0214	32.96	0.0265	40.73
2014	0.0565	0.0147	26.08	0.0173	30.63	0.0245	43.29
2015	0.0487	0.0126	25.85	0.0188	38.63	0.0173	35.52
2016	0.0474	0.0122	25.68	0.0192	40.49	0.0160	33.84
2017	0.0451	0.0118	26.04	0.0151	33.52	0.0183	40.44
2018	0.0530	0.0141	26.7	0.0168	31.67	0.0221	41.64
2019	0.0545	0.0143	26.13	0.0202	36.99	0.0201	36.87

**Table 4:** Markov transition probability matrices of CD&GDD of whole sample and subregions from 2011 to 2019.

	t/(t + 1)	I	II	III	IV
The full samples	I	0.5559	0.4283	0.0157	0.0000
	II	0.0159	0.3651	0.4397	0.1794
	III	0.0000	0.2053	0.4298	0.3649
	IV	0.0000	0.0143	0.2423	0.7435
Eastern region	I	0.4713	0.5223	0.0064	0.0000
	II	0.0055	0.3646	0.3315	0.2983
	III	0.0000	0.1912	0.2941	0.5147
	IV	0.0000	0.0140	0.2009	0.7850
Central region	I	0.6109	0.3774	0.0117	0.0000
	II	0.0000	0.3478	0.5217	0.1304
	III	0.0000	0.2110	0.4726	0.3165
	IV	0.0000	0.0217	0.3370	0.6413
Western region	I	0.5909	0.3831	0.0260	0.0000
	II	0.0308	0.4077	0.4462	0.1154
	III	0.0000	0.1597	0.4202	0.4202
	IV	0.0000	0.0000	0.2706	0.7294
Northeast region	I	0.4627	0.5075	0.0299	0.0000
	II	0.0562	0.3483	0.4382	0.1573
	III	0.0000	0.2821	0.5513	0.1667
	IV	0.0000	0.0333	0.1667	0.8000

**Table 5:** Global spatial correlations of CD&GDD.

Year	W1	W2	Year	W1	W2	Year	W1	W2
2011	0.053 ***	0.358 ***	2014	0.054 ***	0.409 ***	2017	0.064 ***	0.410 ***
	(11.074)	(12.643)		(11.165)	(14.349)		(13.176)	(14.401)
2012	0.046 ***	0.397 ***	2015	0.076 ***	0.410 ***	2018	0.057 ***	0.334 ***
	(9.626)	(13.931)		(15.377)	(14.397)		(11.699)	(11.761)
2013	0.072 ***	0.394 ***	2016	0.086 ***	0.396 ***	2019	0.079 ***	0.371 ***
	(14.729)	(13.936)		(17.317)	(13.927)		(16.088)	(13.061)

Note: *** *p* < 0.01. Z-values are in parentheses.

**Table 6:** Global spatial correlations of CD&GDD in four economic zones.

Region	2011	2012	2013	2014	2015	2016	2017	2018	2019
Eastern region	0.102 ***	0.081 ***	0.120 ***	0.082 ***	0.105 ***	0.124 ***	0.095 ***	0.105 ***	0.129 ***
	(6.306)	(5.178)	(7.514)	(5.224)	(6.500)	(7.588)	(5.967)	(6.527)	(7.842)
Central region	0.059 ***	0.037 ***	0.066 ***	0.034 ***	0.024 ***	0.023 ***	0.008	0.025 ***	0.034 ***
	(5.707)	(3.702)	(5.956)	(3.428)	(2.624)	(2.606)	(1.388)	(2.770)	(3.510)
Western region	0.026 **	0.023 **	0.020 *	0.001	0.019 *	0.025 **	0.030 **	−0.003	−0.009
	(2.239)	(2.087)	(1.952)	(0.938)	(1.863)	(2.177)	(2.397)	(0.715)	(0.405)
Northeast region	−0.030	−0.047	−0.032	0.053 ***	0.036 **	0.009	−0.031	−0.005	0.055 ***
	(0.051)	(0.514)	(0.028)	(2.739)	(2.118)	(1.296)	0.181	(0.847)	(2.721)

Note: * *p* < 0.10; ** *p* < 0.05; *** *p* < 0.01. Z-values are in parentheses.

## Data Availability

Data are available from the sources stated in the text.
